# Endothelin-1 induces cellular senescence and fibrosis in cultured myoblasts. A potential mechanism of aging-related sarcopenia

**DOI:** 10.18632/aging.103450

**Published:** 2020-06-22

**Authors:** Elena Alcalde-Estévez, Ana Asenjo-Bueno, Patricia Sosa, Gemma Olmos, Patricia Plaza, María Ángeles Caballero-Mora, Diego Rodríguez-Puyol, María Piedad Ruíz-Torres, Susana López-Ongil

**Affiliations:** 1 Departamento Biología de Sistemas, Universidad de Alcalá, Alcalá de Henares, Madrid 28871, Spain; 2 Unidad de Investigación de la Fundación para la Investigación Biomédica del Hospital Universitario Príncipe de Asturias, Alcalá de Henares, Madrid 28805, Spain; 3 Instituto Reina Sofía de Investigación Nefrológica (IRSIN) de la Fundación Renal Iñigo Álvarez de Toledo (FRIAT), Madrid 28003, Spain; 4 Area 3-Fisiología y Fisiopatología Renal y Vascular del IRYCIS, Madrid 28046, Spain; 5 Servicio de Geriatría y Unidad de Fragilidad del Hospital de Ciudad Real, Ciudad Real 13003, Spain; 6 Servicio de Nefrología del Hospital Universitario Príncipe de Asturias, Alcalá de Henares, Madrid 28805, Spain

**Keywords:** endothelin-1, fibrosis, senescence, aging, sarcopenia

## Abstract

Endothelial dysfunction, with increased endothelin-1 (ET-1) synthesis, and sarcopenia, characterized by the loss of muscular mass and strength, are two aging–related conditions. However, a relationship between them has not been already established. The aim of this study was to determine whether ET-1 induces senescence and fibrosis in cultured murine myoblasts, which could be involved in the development of sarcopenia related to aging. For this purpose, myoblasts were incubated with ET-1 to assess cellular senescence, analyzed by senescence associated β-galactosidase activity and p16 expression; and fibrosis, assessed by fibronectin expression. ET-1 induced myoblast senescence and fibrosis through ET_A_ receptor. The use of antioxidants and several antagonists revealed that ET-1 effect on senescence and fibrosis depended on ROS production and activation of PI_3_K-AKT-GSK pathway. To stress the *in vivo* relevance of these results, circulating ET-1, muscular strength, muscular fibrosis and p16 expression were measured in male C57Bl6 mice from 5-18-24-months-old. Old mice shown high levels of ET-1 correlated with muscular fibrosis, muscular p16 expression and loss of muscle strength. In conclusion, ET-1 promotes fibrosis and senescence in cultured myoblasts, similar results were found in old mice, suggesting a potential role for ET-1 in the development of sarcopenia related to aging.

## INTRODUCTION

The loss of muscular mass and strength is an inevitable aging related phenomenon, named sarcopenia. The mechanisms involved in its origin and progression remain to be clearly elucidated. Malnutrition, decreased physical exercise and the decline in sex steroid hormone production and in insulin sensitivity play an important role in sarcopenia [1]. Sarcopenic muscles suffer profound changes in their architecture, exhibiting both reduced fiber size and reduced fiber number, especially of the type II fibers, reduced number of satellite cells and accumulation of connective tissue and fat between the fibers [2–4].

Tissue fibrosis is characterized by the excessive deposition of extracellular matrix (ECM) proteins, including collagens and fibronectin (FN) [5]. Fibrosis is triggered by the action of several growth factors, such as TGF-ß1 and CTGF, by fibroblasts activation and by the inflammation process [6, 7]. Progression of chronic diseases in parenchyma organs such as liver, kidney, heart, and lung is associated with fibrosis, and the fibrotic process plays a critical role in the detrimental damage of these organs [6]. In fact, the appearance of fibrotic areas in skeletal muscle has been extensively studied in skeletal muscular dystrophy diseases and has been associated with the loss of muscular strength [8]. In skeletal muscle, fibrosis can result from either acute muscle trauma or chronic injury conditions, with excessive accumulation of ECM components [9]. Muscular fibrosis has been related to the activation of resident fibroblasts [10] or may also be caused by a faulty muscle-repair process initiated by the activation of muscle satellite cells [11]. After damage, satellite cells are activated into proliferating myoblasts that fuse to form new myofibrils. However, in the aged muscle, the regenerative cascade is characterized by a shift in the functional myofibril repair leading to an increase in ECM [12].

One of the mechanisms involved in the decline of satellite cell function in aged muscle is cellular senescence [3, 13], defined by Hayflick as an irreversible arrest of cell division [14]. Cellular senescence can be induced prematurely by activated oncogenes, DNA damage and oxidative stress [15, 16] as well as by extracellular systemic factors such as hyperosmolarity, high glucose [17] and glycated albumin [18]. In this sense, we have recently described that elevated extracellular phosphate concentration induced senescence in cultured myoblasts, disrupting the autophagy mechanism through ILK overexpression [19].

On the other hand, an additional aging-related phenomenon is endothelial dysfunction. A decreased production of nitric oxide together with an increased release of endothelin-1 (ET-1) has been described in some aging-related cardiovascular diseases [20]. ET-1 is a potent vasoconstrictor peptide having mitogenic and proliferative activity [21]. ET-1 plays a pivotal role in vascular remodeling inducing the synthesis of different ECM components [22], induces proliferation and migration of smooth muscle cells [23], and also the fibrotic process [24]. Biological actions of ET-1 necessarily involve the activation of specific G-protein coupled receptors, ET_A_ and/or ET_B_, which are present not only in vascular cells but also in numerous tissues including lung, heart, kidney, intestine, adrenal gland, eye, and brain [25]. High levels of ET-1 have been found in fibrotic diseases affecting lung, kidney, heart, or skin [24]. However, the role of ET-1 in the development of skeletal muscle fibrosis has not been described before.

The relationship between ET-1 and aging has begun to be studied lately. Wang et al. found that fibroblasts isolated from the aged mice hearts and also in aged human hearts expressed high levels of ET-1, suggesting that aging-related cardiac fibrosis was, at least partially, dependent on the upregulation of ET-1 [26]. ET-1 also contributes to the fibrotic phenotype, inducing the expression of collagen, FN and CCN2 in fibroblasts isolated from fibrotic lesions [27]. Moreover, ET-1 has been reported to induce cellular senescence in cultured vascular endothelial cells [28].

We hereby propose that ET-1 increases with age and promotes synthesis of ECM proteins and senescence of myoblasts, leading to muscular fibrosis and contributing to the aging-related loss of muscle force.

## RESULTS

### Endothelin-1 induces senescence and increases fibronectin expression in cultured murine myoblasts (C_2_C_12_) through ET_A_ receptor

**Figure 1:**
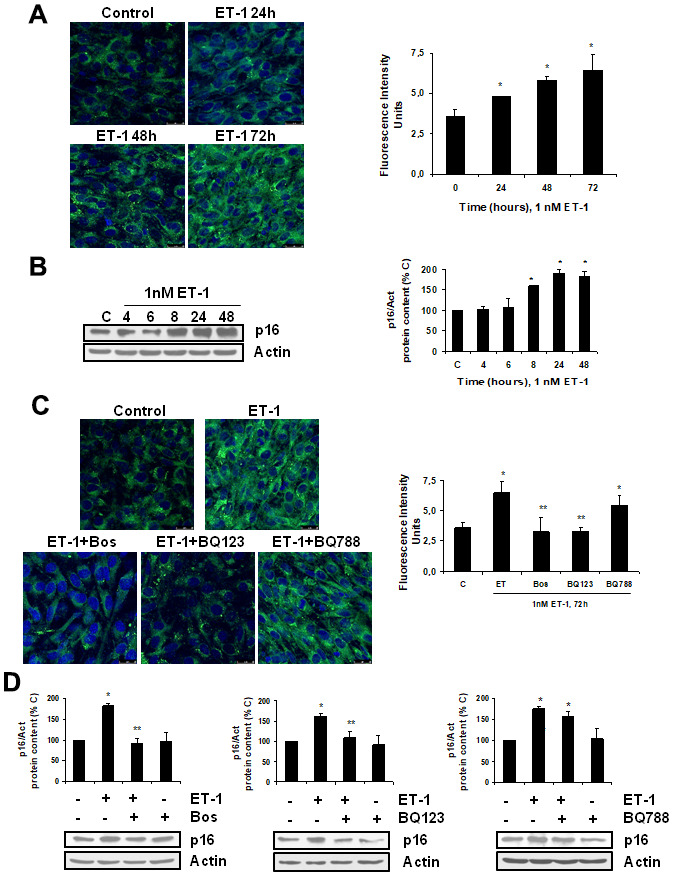
**Endothelin-1 induces senescence in mouse myoblasts (C_2_C_12_) through ET_A_ receptor.** Cells were grown on coverslips (**A**, **C**) and incubated with 1 nM ET-1 at different times (**A**, **B**), or incubated with 10 μM Bosentan (Bos), 100 nM BQ-123 (BQ123) or 100 nM BQ-788 (BQ788) added 30 min before ET-1 (1 nM), and then incubated for 72h (**C**) or 48h (**D**). Then, senescence was tested measuring SA-ß-GAL activity (panel **A**, **C**) and protein content from p16 (panel **B**, **D**). Representative microphotographs are shown on the left with 40x magnification and the densitometric analysis is shown on the right panel **A**, **C**. Scale bar, 50 μm. A representative Western blot of p16 is shown next to the densitometric analysis on the panel **B**, **D**. Values are the mean±SEM of 6 independent experiments, *p<0.05 vs. control cells (**C** or time 0), and **p<0.05 vs ET alone.

**Figure 2:**
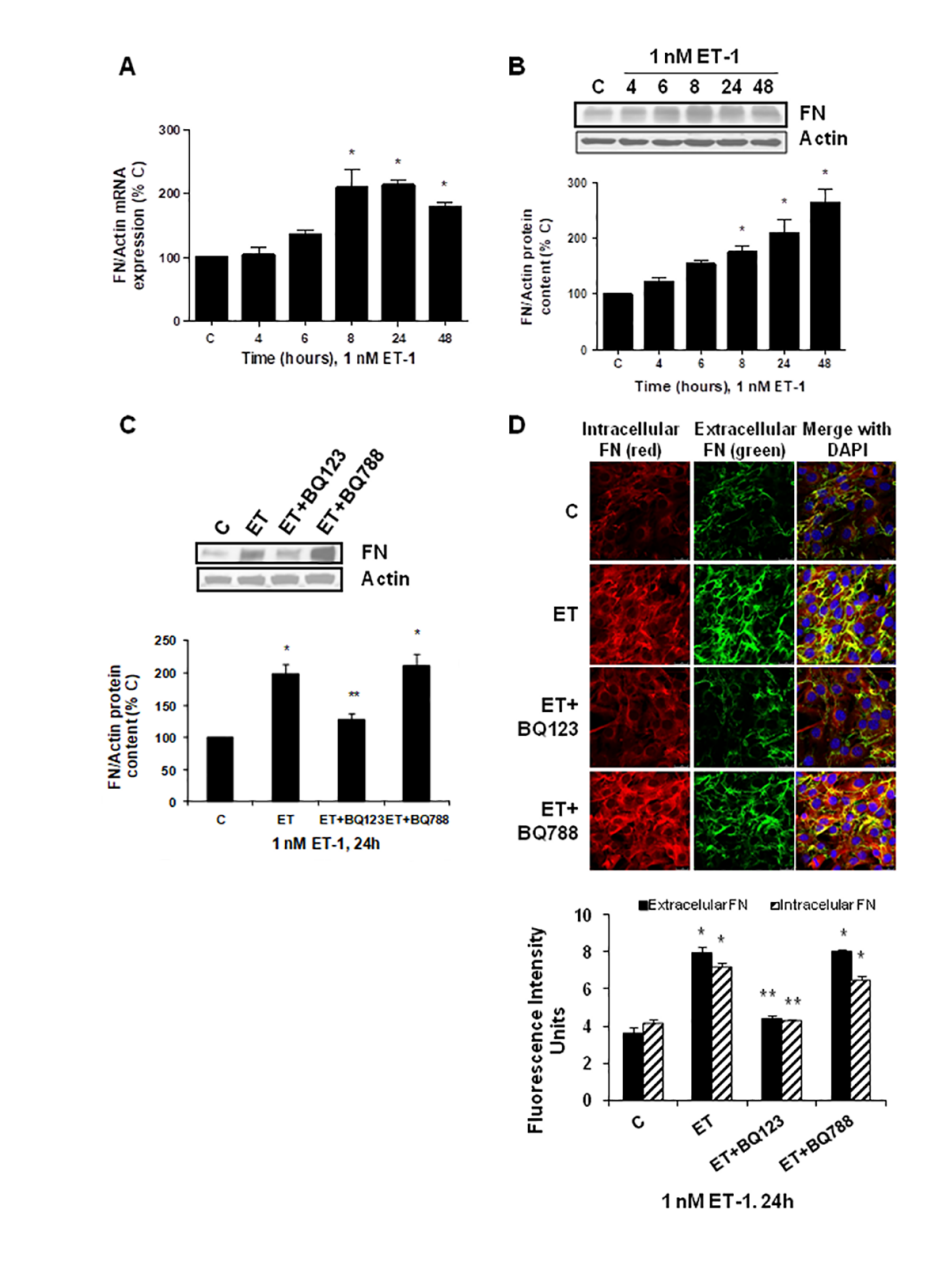
**Endothelin-1 increases FN expression in mouse myoblasts (C_2_C_12_) through ET_A_ receptor.** Cells were incubated with 1 nM ET-1 at different times. Then, FN mRNA expression was assessed by RT-qPCR (panel **A**) and FN protein content by Western blot (panel **B**). To study the ET receptor implicated, cells were incubated with 100 nM BQ-123 (BQ123) or 100 nM BQ-788 (BQ788) added 30 min before ET-1 (1 nM), and then incubated for 24h. Then, FN protein content (panel **C**) as well as intracellular FN (in red) and extracellular FN (in green) expression (panel **D**) were studied by Western and immunofluorescence, respectively. In the experiments of the analysis of protein content, a representative Western blot is shown at the top with the densitometric analysis below (panel **B,** **C**). Values are the mean±SEM of 6 independent experiments, *p<0.05 vs. control cells (**C**), and **p<0.05 vs ET alone.

To explore the effect of ET-1 on C_2_C_12_, we first confirmed that cultured myoblasts expressed specific ET-1 receptors. The expression of ET-1 receptors in cultured myoblast was confirmed by western blot and real time-PCR. C_2_C_12_ expressed both types of ET receptors, more ET_A_ than ET_B_ receptors (Supplementary Figure 1), compared with endothelial cells. Then, we tested whether ET-1 induced cellular senescence and extracellular matrix protein expression in myoblast cells. For this purpose, C_2_C_12_ were incubated with 1 nM ET-1 at different times to assess cellular senescence by measuring senescent-associated β galactosidase activity (SA-β-GAL) and by p16 protein expression. ET-1 induced senescence in C_2_C_12_ in a time-response way (Figure 1A, 1B), reaching a peak at 72 h in SA-ß-GAL activity and at 48h in p16 expression. The effect was mediated by ET_A_ receptor as it was blocked in the presence of the dual ET_A/B_ receptor antagonist (Bosentan) and of the specific ET_A_ receptor antagonist (BQ123), but not in the presence of the specific ET_B_ receptor antagonist (BQ788) (Figure 1C, 1D). Proliferation of myoblasts after treatment with ET-1 was checked by expression of proliferating cell nuclear antigen (PCNA) by western blot assays. Cells treated with ET proliferated less than control cells, suggesting that myoblasts underwent a growth arrest upon ET treatment (Supplementary Figure 2). FN expression was also measured after ET-1 addition to evaluate potential fibrosis at different times, as FN is an important ECM protein. ET-1 was able to induce FN expression, not only at mRNA levels (Figure 2A) but also at protein levels (Figure 2B) after 8 h incubation, reaching a maximum effect between 8-24 h. This effect was also mediated by ET_A_ receptor as it was blocked in the presence of the specific ET_A_ receptor antagonist (BQ123), but not of the specific ET_B_ receptor antagonist (BQ788) assessed by both western blot and immunofluorescence staining (Figure 2C, 2D). Figure 2D shows as ET-1 induced, not only intracellular FN expression in red, but also extracellular FN expression in green. Both of them are mediated by ET_A_ receptor.

**Figure 3:**
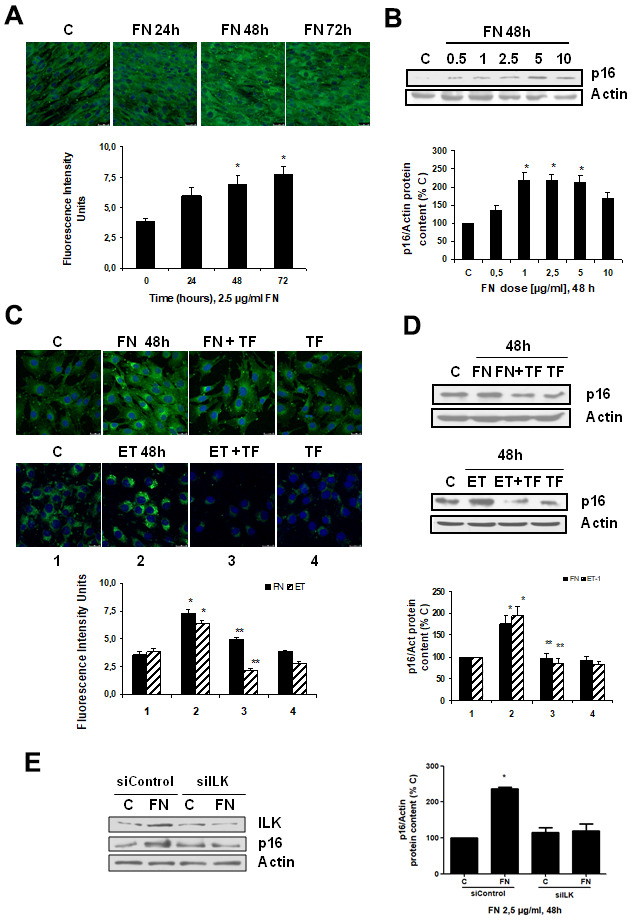
**Fibronectin induces senescence in mouse myoblasts (C_2_C_12_) through integrin/ILK activation.** Cells were grown on coverslips (panels **A**, **C**) to test senescence measuring SA-ß-GAL activity and p16 protein content by Western blot (panels **B**, **D**). (**A**) Cells were incubated with 2.5 μg/ml FN at different times to assess SA-ß-GAL activity by confocal microscopy. (**B**) Cells were incubated at different doses of FN for 48h to analyze p16 protein content. (**C**) Cells were incubated with 2.5 μg/ml FN or 1 nM ET-1 in the presence or not of 50 μM Tirofiban (TF) for 48h to assess senescence by SA-ß-GAL activity (panel **C**) or by p16 protein content (panel **D**). Representative microphotographs are shown at the top with 40x magnification and the densitometric analysis is shown below. Scale bar, 50 μm. A representative Western blot of p16 is shown at the top and the densitometric analysis is shown below. In panels **C** and **D** closed bars represent data of FN treatment and stripped bars represent data of ET-1 treatment; lane 1: control cells; lane 2: FN or ET alone; lane 3: FN or ET plus TF; lane 4: TF alone. Values are the mean±SEM of 6 independent experiments, *p<0.05 vs. control cells (**C** or time 0), and **p<0.05 vs ET or FN alone. (**E**) Cells were transfected with siRNA against ILK or scrambled as siControl to assess senescence by p16 protein content upon 2.5 μg/mL FN treatment for 48h. A representative Western blot of ILK and p16 are shown on the left panel and the densitometric analysis is shown on the right. Values are the mean±SEM of 3 independent experiments, *p<0.05 vs. control cells (**C** from siControl).

To analyze whether there was a link between fibrosis and cellular senescence, we evaluated the effect of FN itself at different times and doses on cellular senescence. FN was able to induce senescence on myoblasts just as ET-1 did, which was evaluated by measuring SA-β-GAL activity and p16 protein content (Figure 3A, 3B), reaching a peak with FN 2.5 μg/ml at 48 h. Tirofiban (TF, 50 μM), a peptide preventing the effect of FN, blocked the effects of FN and ET-1 on senescence (Figure 3C, 3D), suggesting that ET-1 effect on myoblast senescence was related to the increase in FN expression. Additional experiments to silence ILK were done to check if the effect of FN on senescence is mediated by ILK activation. Results suggest that ILK could mediate FN-induced senescence as cells without ILK did not increase p16, whereas cells with ILK showed p16 overexpression under FN treatment (Figure 3E).

### Endothelin-1 effect on senescence and fibronectin expression depends on ROS production and on the activation of PI_3_K-AKT-GSK pathway through ET_A_ receptor

To examine the intracellular mechanisms involved in ET effect on senescence and fibronectin expression, we studied the reactive oxygen species (ROS) production and also the role of PI_3_K-AKT-GSK pathway.

**Figure 4:**
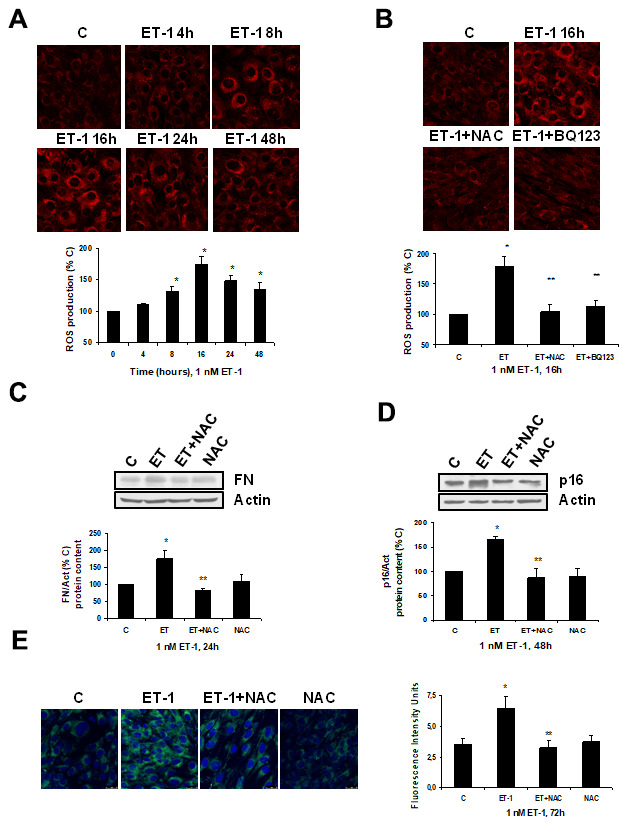
**Role of ROS in endothelin-dependent fibrosis and cellular senescence.** (**A**, **B**) Cells were incubated with 1 nM ET-1 at different times, some cells were incubated in the presence of 100 μM N-acetylcysteine (NAC) or 100 nM BQ123, and then 1 nM ET-1 was added and incubated for 16h (**B**). CellROX probe was added during the last 30 min of incubation. After being washed twice, *in vivo* cells were visualized by microscopy confocal to test ROS production in red. Representative microphotographs are shown at the top with 40x magnification, scale bar, 50 μm. The densitometric analyses are shown below. (**C**–**E**) Cells were incubated with 1 nM ET-1 in the presence of 100 μM NAC to assay FN protein expression by Western blot (**C**). Cellular senescence was assessed by measuring p16 protein content for 48h by Western blot (panel **D**) and SA-ß-GAL activity for 72h (panel **E**). A representative Western is shown on the top with the densitometric analysis below (panels **C**, **D**). Representative microphotographs are shown on the left panel **E** with 40x magnification and the densitometric analysis is shown on the right. Scale bar, 50 μm. Values are the mean±SEM of 6 independent experiments, *p<0.05 vs. control cells (**C** or time 0), and **p<0.05 vs ET alone.

Firstly, ET-1 was able to induce ROS production (Figure 4A), reaching a maximum peak around 8-16 h, and it was completely blocked in the presence of the antioxidant N-acetylcysteine (NAC) and also of the specific ET_A_ receptor antagonist (BQ123) (Figure 4B). ROS production also seems to be involved in the mechanism of action of ET-1, because the use of NAC blocked not only the FN expression (Figure 4C), but also the cellular senescence, measured as SA-ß-GAL activity (Figure 4E) and as p16 protein expression (Figure 4D).

**Figure 5:**
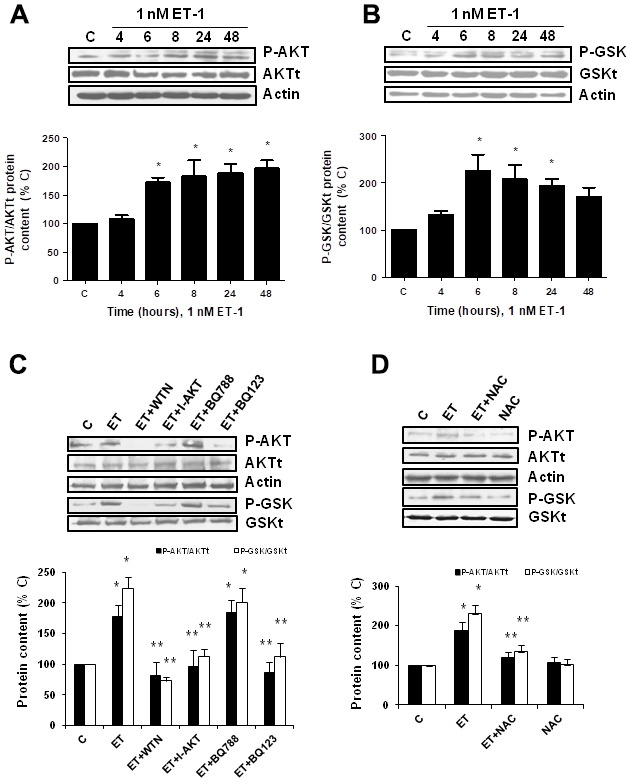
**Endothelin-1 induces activation of PI_3_K-AKT-GSK pathway in mouse myoblasts (C_2_C_12_) through ET_A_ receptor and ROS production.** (**A**, **B**) Cells were incubated with 1 nM ET-1 at different times. Activation of PI_3_K-AKT-GSK pathway was analyzed by Western blot measuring phosphorylation of AKT (P-AKT, panel **A**) and phosphorylation of GSK (P-GSK, panel **B**). (**C**, **D**) Cells were incubated in the presence of different antagonists to block PI_3_K-AKT-GSK pathway (Wortmannin: 10 μM WTN; AKT inhibitor: 30 μM I-AKT), to block ET receptors (ET_A_ receptor antagonist: 100 nM BQ123; ET_B_ receptor antagonist: 100 nM BQ788) (**C**), and to block ROS production (antioxidant N-acetylcysteine: 100 μM NAC) (**D**). All of them were added at least 30 min before 1 nM ET-1 for 24h, to assay P-AKT (closed bars) or P-GSK (open bars). Representative Western blots are shown at the top of each panel. The densitometric analysis is shown below of each panel. Values are the mean±SEM of 5 independent experiments, *p<0.05 vs. control cells (**C**), and **p<0.05 vs ET alone.

**Figure 6:**
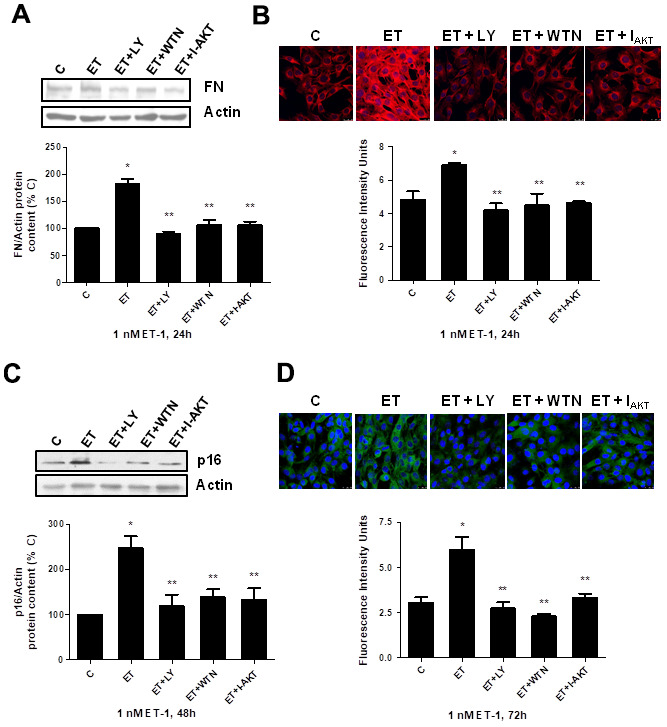
**Role of PI_3_K-AKT-GSK pathway in endothelin-dependent cellular fibrosis and cellular senescence.** Cells were incubated in the presence of different antagonists to block PI_3_K-AKT-GSK pathway (Wortmannin: 10 μM WTN; LY-294,002 hydrochloride: 50 μM LY; AKT inhibitor: 30 μM I-AKT). All of them were added at least 30 min before adding 1 nM ET-1 for 24h to assay FN expression by Western blot (panel **A**) or by Immunofluorescence (panel **B**), and to assay senescence by measuring p16 protein content for 48h by Western blot (panel **C**) and SA-ß-GAL activity for 72h (panel **D**). Representative Western blots are shown at the top of panels (**A**, **C**). Representative microphotographs are shown on the top of panel **B**, **D** with 40x magnification, scale bar, 50 μm. The densitometric analysis is shown below of each panel. Values are the mean±SEM of 6 independent experiments, *p<0.05 vs. control cells (**C**), and **p<0.05 vs ET alone.

Secondly, the role of PI_3_K-AKT-GSK was assessed since other authors proposed it as a pathway implicated in pro-fibrotic actions of ET-1 [29]. ET-1 induced AKT and GSK phosphorylation without changing the expression of total AKT or total GSK proteins (Figure 5A, 5B), confirming that ET-1 induces activation of this pathway in our cells. Then, different antagonists were added to block PI_3_K-AKT-GSK pathway such as wortmannin (WTN, 10 μM) which blocks phosphatidylinositol 3-kinase (PI_3_K) preventing phosphorylation and activation of AKT; and the AKT inhibitor (I-AKT, 30 μM), which blocks phosphorylation of GSK-3ß. In addition, specific antagonist of ET_A_ (BQ123, 100 nM) and ET_B_ (BQ788, 100 nM) were used to confirm that the effect was mediated by ET_A_ receptor and not by ET_B_ receptor. All antagonists except BQ788 were able to block phosphorylation of AKT and GSK induced by ET-1 1 nM for 24 h (Figure 5C). The use of NAC also reduced the phosphorylation of AKT and GSK proteins (Figure 5D), suggesting that ROS are implied in the activation of PI_3_K-AKT-GSK pathway. FN expression and senescence induced by ET-1 was assessed in the presence of WTN, I-AKT and also LY-294,002 hydrochloride (LY, 50 μM) which blocks phosphatidylinositol 3-kinase (PI_3_K) preventing phosphorylation and activation of AKT. All of them blocked not only the ET-1 effect on FN expression studied by Western blot (Figure 6A) and by immunofluorescence (Figure 6B), but also the effect of ET-1 on senescence studied by p16 protein expression (Figure 6C) and by SA-ß-GAL activity (Figure 6D). These results point to ET-1-induced ROS could activate PI_3_K-AKT-GSK pathway and then stimulate FN expression and myoblast senescence.

Fibrosis is often the consequence of inflammation and inflammation is known to trigger senescence in cells and tissues. The effect of ET-1 on the expression of several cytokines such as TNF-alfa, IL-6 and MCP-1 was evaluated by real time PCR. ET-1 induced expression of these cytokines at short times, returning to baseline after 16h, suggesting that ET-1 might induce inflammation in the first place (Supplementary Figure 3), which could be involved in development of fibrosis and senescence induced by ET-1.

### Aged mice showed high levels of circulating ET-1 and muscular fibrosis

**Figure 7:**
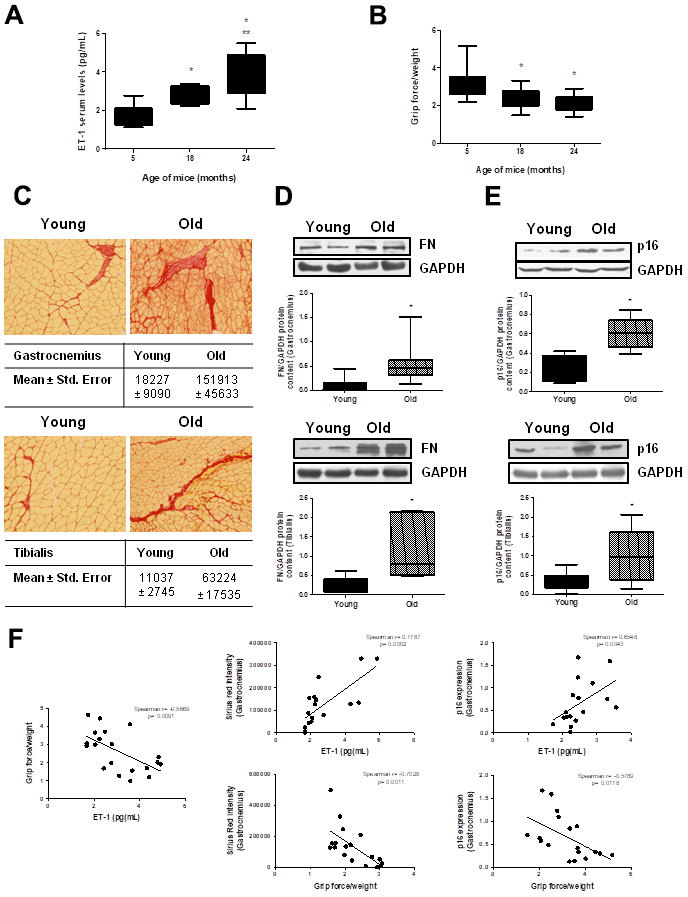
**Aging mice present high circulating ET-1 levels, loss of muscle strength, fibrosis and senescence in gastrocnemius and tibialis anterior muscles.** Animals were kept on a 12:12h light-dark cycle, at 24°C, and food and water were available ad libitum. Male C57Bl6 mice from 5, 18, and 24 month-old were used, 10 animals per group. (**A**) Serum ET-1 levels was measured by ELISA. (**B**) Muscle force was registered using the 4 limb grip test and data were corrected by body weight of each mouse. Values are the mean±SEM of 10 mice, *p<0.05 vs. 5-month-old; **p<0.05 vs 18 month-old. Mice of 5-month-old (Young-closed bars) and 24-month-old (Old-stripped bars) were used to measure Sirius red staining in sections of the gastrocnemius (GNM) and the tibialis anterior muscles to visualize fibrosis (**C**), to analyze FN protein expression by Western blot (**D**) and to assess senescence by measuring p16 protein content by Western blot (**E**) in those muscles of the same mice. (**C**) Sirius red staining (20x) is shown with the mean ± standard error below pictures. (**D**, **E**) A representative Western blot was shown above with the densitometric analysis below. Values are the mean±SEM of 20 mice, *p<0.05 vs. young mice. (**F**) Graphs of correlations based on data from young (5-month-old) and old (24-month-old) mice were shown: ET-1 levels and grip force (Spearman r= -0.5669, p= 0.0091), ET-1 levels and Sirius red in GNM (Spearman r= 0.7787, p= 0.0002), ET-1 levels and p16 expression in GNM (Spearman r= 0.6548, p= 0.0043), grip force and Sirius red in GNM (Spearman r= -0.7028, p= 0.0011) and grip force and p16 expression in GNM (Spearman r= -0.5789, p= 0.0118).

To stress the *in vivo* relevance of these results, ET-1 serum levels were measured in male C57Bl6 mice at different ages: 5, 18 and 24 months-old. Results showed that ET-1 levels increased with age, reaching a maximum at 24 months (Figure 7A). Muscle force was measured by the 4 limb grip strength test on the same animals, finding an age-related significant reduction (Figure 7B). Then, histological analysis to look for the appearance of muscular fibrosis was performed in the gastrocnemius and the tibialis anterior muscle on the same animals using Sirius Red staining. In addition, FN protein expression was also analyzed by Western blot in those muscles. Figure 7 shows representative experiments from young mice from 5-month-old compared with old mice from 24-month, showing more fibrotic areas in old mice than in young mice (Figure 7C) and a significant increase in FN protein content in gastrocnemius and tibialis anterior muscle from 24-month mice compared with young mice (5-month) (Figure 7D). Furthermore, p16 protein expression was also evaluated by western blot in the gastrocnemius and the tibialis anterior muscles, finding a significant increment in old mice versus young mice (Figure 7E), suggesting a potential senescence in aged muscles. Figure 7F shows different correlation data in the gastrocnemius muscle, there are a significant negative correlation between muscular strength from mice measured with grip test and their levels of ET-1, muscular fibrosis measured as Sirius red staining and senescence evaluated by p16 protein content. Additionally, there are a significant correlation between levels of ET-1 and the appearance of muscular fibrosis and senescence.

**Figure 8:**
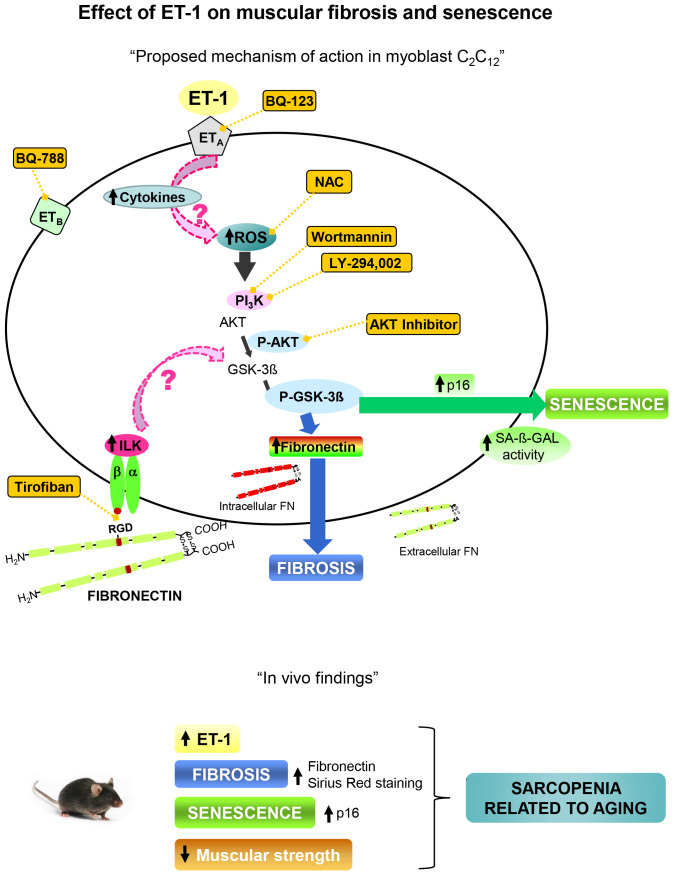
**Proposal mechanism of action of ET-1 on muscular fibrosis and senescence.** Myoblast cells present both type of ET-1 receptors, ET_A_ and ET_B_, which are inhibited by specific antagonists such as BQ123 and BQ788, respectively. The binding of ET-1 to ET_A_ receptor induces fibrosis and senescence through ROS production by activation of PI_3_K-AKT-GSK pathway. The inflammation induced by ET-1 could be also implied. Several antagonists were used to inhibit both, ROS production with the antioxidant N-acetylcysteine (NAC), and the PI_3_K-AKT-GSK pathway with AKT inhibitor, LY-294,002 and Wortmannin, to check the mechanism. Fibronectin could induce senescence through integrin receptor activation by joining to RGD sequence, and then trigger some downstream pathways through ILK activation. Tirofiban blocks the joining of FN to RGD sequence. Findings in aged mice are showed below which are similar to those found in myoblast cells induced by ET-1, suggesting that the appearance of fibrosis and senescence could be involved in the genesis of sarcopenia related to aging. Note: all antagonists or inhibitors are represented in an orange box and unexplored mechanisms with an arrow with dashed pink line.

Present results point to significance role of ET-1 in the regulation of pathophysiological processes related with aging such as fibrosis, senescence and decline of force, which could be involved in the development of sarcopenia. A proposal mechanism of action of ET-1 on muscular fibrosis and senescence is shown in Figure 8.

## DISCUSSION

Aging is characterized by the progressive loss of function in different tissues which can provoke, among others, endothelial dysfunction, with increased ET-1 synthesis, ECM accumulation in different tissues, and sarcopenia. ET-1 has been associated to the development of fibrosis in different cell and tissue contexts [22, 24, 27, 29], even it can induce cellular senescence in human endothelial cells [28], but the role of ET-1 in skeletal muscle aging has never been described before. For this reason, we decided to study its effects on cultured muscle cells using a well-described experimental cellular model; mouse myoblast C_2_C_12_ cells [30]. We analyzed the effect of ET-1 on some of the best studied aging-related aspects of myoblast biology, in particular on cellular senescence and ECM protein synthesis, since aging is associated with reductions in both muscle stem cell function and muscle regeneration potential, and with increased muscular fibrosis [4, 31].

ET-1 receptors are expressed in many organs and tissues, even they were described in C_2_C_12_ cells by Tsui et al. [32]. According with that, we found both types of receptors, ET_A_ and ET_B_, in skeletal muscle and in muscle tissues, which were analyzed by Western blot and by qPCR. C_2_C_12_ expressed both types of receptors, ET_A_ and ET_B_, with the ET_A_ being the predominant one. In contrast, the ET_B_ was the predominant receptor on endothelial cells. Besides ET_A_ receptor has been preferentially involved in the pro-fibrotic effects of ET-1 [33].

We have previously described that cultured myoblast undergo cellular senescence after exposition to high extracellular phosphate concentration, compromising the regenerative capacity of the muscle [19]. In addition, hyperphosphatemia, which dramatically affects the behavior of vascular cells [34], increases ECE-1 activity in endothelial cells, which induces the synthesis of ET-1 and also senescence in endothelial cells [28]. The role of ET-1 on cellular senescence has not been previously evaluated in other cellular types out of blood vessels, but now, we hereby confirm that ET-1 also induced senescence in myoblasts. This effect was mediated by ET_A_ receptor because the use of a specific ET_A_ receptor antagonist (BQ123) or the unspecific dual ET_A/B_ receptor antagonist (Bosentan) blocked the effect, whereas the use of a specific ET_B_ receptor antagonist (BQ788) did not modify ET-1 effect on senescence. Cell senescence was analyzed by p16 protein expression and SA-β-GAL activity as previously described [35], without changing in p53 protein expression (data not shown). The senescence of myoblast has been associated with muscular dystrophy and with premature muscle wasting occurring in Bmi1 deficient young mice [36]. Senescence induced by ET-1 at vascular level has been involved in some pathologies [37], such as diabetes [38], varicosity [39] and thrombosis [40]. So, senescence induced by ET-1 at muscle level could be implicated in the development of fibrosis, exacerbating muscle damage, as the regenerative potential of skeletal muscle declines with age and this impairment is associated with an increase in tissue fibrosis [4, 31]. Brack et al. showed that muscle stem cells (satellite cells) from aged mice tend to convert from a myogenic to a fibrogenic lineage as they begin to proliferate and increase tissue fibrosis [4]. In fact, myoblast cells under ET-1 treatment increased significantly FN expression in an ET_A_ receptor-dependent way.

Interestingly, it is the first time that the potential role of FN on ET-1-induced senescence has been demonstrated. FN itself was able to induce mouse myoblast senescence and Tirofiban, an integrin αIIbß3 antagonist [41] which blocks the Arg-Gly-Asp (RGD)-enriched fragment of FN, prevented cell senescence induced by both ET-1 and FN. This result suggests that FN can be mediating the effect of ET-1 on myoblast senescence through integrin activation, by an unknown mechanism which needs more studies. According with us, some authors suggested that the integrin signaling can contribute not only to senescence but also fibrosis effects, for instance, cellular senescence caused by deficiency of retinoic acid inducible gene-I (Rig-I) through amplifying integrin ß3/p38 MAPK signaling [42]; and bleomycin-induced fibrosis initiated through integrin activation and imbalance in the redox state of the cell [43].

In this regard, we analyzed ILK, a well-known protein linked to integrin activity. Likewise, when ILK was silenced in C_2_C_12_, FN did not increase p16 expression, suggesting that ILK activation might be implied in the senescence induced by FN. Briefly, the mechanism could involve the integrin/ILK activation through extracellular FN which could activate some downstream signaling pathway such as p38/ErK/MAPK or even PI_3_K-AKT-GSKβ [42–45], causing senescence. However, further studies to evaluate the underlying mechanism behind FN-induced senescence are needed.

To study the mechanisms involved in ET-1 effects on fibrosis and senescence of myoblast; ROS production and PI_3_K/AKT/GSK pathway activation were evaluated. In sarcopenia, an increase in mitochondrial ROS production together with impaired mitochondrial function and oxidative damage [46] has been described. As in other aging-related conditions [28, 47], higher levels of ROS seem to be involved in ET-1-induced myoblast senescence because the effect disappeared in the presence of NAC. NAC is an antioxidant that acts replenishing intracellular cysteine levels, which are essential for glutathione formation, responsible for detoxify hydrogen peroxide [48]. Furthermore, NAC also prevented FN expression induced by ET-1 treatment. Present results suggest that ROS production induced by ET-1 mediated fibrosis and senescence found in mouse myoblast cells.

Taking to account that fibrosis is often the consequence of inflammation and inflammation is known to trigger senescence in cells and tissues, our results suggest that the inflammation induced by ET-1 could trigger fibrosis through activation of ROS production and the induction of PI_3_K/AKT pathway, as well as, the senescence found in myoblasts.

Next, as it has been reported that ET-1 induces PI_3_K/AKT pathway, which is responsible for inhibiting fibroblast apoptosis [49], protecting ovarian carcinoma cells against paclitaxel-induced apoptosis [50], or even regulating ET-1-induced fibroblast differentiation and contraction [29], we investigated the role of PI_3_K/AKT/GSK pathway activation by ET-1 in the regulation of FN expression and in the activation of senescence. ET-1 induces phosphorylation of AKT and GSK, suggesting that ET-1 activates PI_3_K/AKT/GSK pathway, like other authors have reported [29, 49]. PI_3_K/AKT/GSK activation depends on ET_A_ receptor because it was completely blocked by the specific ET_A_ receptor antagonist BQ123 but not by the ET_B_ receptor antagonist BQ788. FN expression depends on this pathway as specific inhibitors of PI_3_K/AKT/GSK signaling were able to block ET-1 effect. Furthermore, the activation of senescence induced by ET-1 was also blocked in the presence of these inhibitors. Finally, we confirmed that ROS production was involved in the activation of PI_3_K/AKT pathway, as the use of NAC completely blocked the phosphorylation of AKT and GSK induced by ET-1.

To stress the *in vivo* relevance of these results, we study the potential relationship between ET-1, muscular fibrosis, senescence and the decline in muscle force in old mice. In a previous work, we described that concentration of ET-1 in serum was elevated in 20-month-old mice [28] as a consequence of an increased expression of endothelin-converting enzyme-1 (ECE-1) in aorta. Now, we have performed a more exhaustive analysis by measuring ET-1 levels in serum from mice at different ages: 5, 18 and 24 months old. In fact, ET-1 was progressively increasing with advanced age, reaching the maximum peak at 24 months. Elevated circulating level of ET-1 has also been reported in aged humans and activation of ET-1 system has been related to aging-associated diseases, such as diabetes, atherosclerosis, hypertension and cancer [51]. Moreover, experimental models of premature aging, such as the telomerase-deficient mice, have shown increased circulating ET-1 as a result of the increment in ECE-1 expression [52]. However, there have been no studies demonstrating the role of ET-1 in the aging of skeletal muscle so far.

Together with circulating ET-1, muscle strength was evaluated in the same mice, finding a significant reduction in the muscle force when the mice's age was advancing. Interestingly, those mice that had higher levels of ET-1 had less muscle strength, suggesting a relationship between both of them. ET-1 is one of the best known pro-fibrotic factors and aging-associated changes in muscle structure include the appearance of fibrotic areas between the fibers, which can be directly involved in the decline of muscular function [53]. We found that muscles isolated from old mice (24-month-old) showed larger fibrotic areas than those isolated from young mice (5-month-old) assessed by Sirius red staining, which stains mainly collagen fibers. Furthermore, FN protein expression was also evaluated in the gastrocnemius and tibialis anterior muscles from those mice, finding an increase in old mice compared with young mice. Both FN and collagen are considered proteins of ECM, which can be accumulated in muscle causing fibrosis. Again, we checked whether those mice with more muscular fibrosis had a minor muscular strength and a higher concentration of ET-1 in serum, since the intensity of Sirius red staining was negatively correlated with muscle force and positively correlated with circulating ET-1. In addition, p16 protein expression was assessed by western blot in the same muscles from those mice, finding also an increased expression of p16 in old mice compared to young ones.

Baker et al. confirmed the role of senescent cells in some aged-related diseases [54, 55], where the presence of senescent cells within a tissue was related to the loss of function, and treatment lead to remove senescent cells from tissues improved the organ function. However, they did not confirm whether muscle function was also improved with that treatment. In this sense, we found a negative correlation between grip test and p16 expression in mice, suggesting that the presence of senescence cells in gastrocnemius are linked to loss of strength. These results allow us to hypothesize that circulating ET-1 is increased with age and reaches skeletal muscle cells, where it can promote fibrosis and senescence determining the loss of muscular strength characteristic of aging. In this sense, some authors found a link between ET-1 and aging-related cardiac fibrosis; Wang et al. demonstrated that ET-1 increased FN and collagen expression in cardiac fibroblast, and hearts from old mice showed cardiac fibrosis with upregulation of ET-1 [26]. Meyer et al. observed that aging increased mRNA myocardial expression of ECE-1 and ET receptors in mice, which could be associated with heart failure, fibrosis and left ventricular hypertrophy [56]. According to this data, the fibrosis we found in the gastrocnemius and tibialis anterior muscles from old mice could explain at least in part the reduction in muscle force. Raz et al. suggested that chronic age-associated muscle degeneration was associated with higher fibrosis in muscle [31] and the relationship between muscular fibrosis and loss of strength has also been described in several muscular dystrophies [57]. With respect to senescence increased in old mice, previous results from our group [19, 45] are in agreement, as they found that old mice had high levels of serum phosphate linked to increased expression of ILK and senescence genes.

In summary, we demonstrated that ET-1-induced senescence and fibrosis in cultured mouse myoblast cells depends on the increase of extracellular FN, that interacting with integrin receptor, induces ILK expression, ROS production and activation of PI_3_K-AKT-GSK pathway. Also, we provided solid evidences for the *in vivo* relevance of these results since aged mice showed not only a higher expression of FN and p16 in the gastrocnemius and tibialis anterior muscles but also high levels of serum ET-1, which are negatively correlated with muscle force and positively correlated with Sirius red staining. High ET-1 levels related to aging could increase senescent cells and muscular fibrosis, which could be involved in impairing muscle function decline. We hereby propose that levels of circulating ET-1 increase with age, contributing to the appearance of fibrosis and senescence in the gastrocnemius and tibialis anterior muscles, which could promote the loss muscular strength related to aging.

## MATERIALS AND METHODS

### Materials

Culture plates, culture media, blueStar-prestained protein marker, BCA protein assay reagent, nitrocellulose membrane, secondary horseradish peroxidase-conjugated goat anti-mouse IgG, CL-Xposure films were from Cultek (Thermo Fisher Scientific brand, Madrid, Spain), and Supersignal West Pico detection system was from Pierce (Thermo Fisher Scientific brand, Madrid, Spain). The ET-1 ELISA system was from Immuno-Biological Laboratories (IBL Co., Japan). Rabbit polyclonal anti-Fibronectin antibody (ab6584), rabbit monoclonal anti-p16 antibody (ab51243) and Picro Sirius Red Stain kit (ab150681) were from Abcam (Cambridge, UK). Rabbit phospho-AKT (Ser473) antibody (9271), rabbit AKT antibody (9272), rabbit phospho-GSK-3ß (Ser9) antibody (9336), rabbit GSK-3ß antibody (9315) and rabbit ILK antibody (3862) were from Cell Signaling (IZASA, Barcelona, Spain). Rabbit antibodies against ET receptors ET_A_ (anti-Ednra-MBS243959) and ET_B_ (anti-Ednrb-MBS2523107) were from MyBioSource (San Diego, CA, USA). Acrylamide-bisacrylamide was from Hispanlab-Pronadisa (Madrid, Spain). Electrophoresis equipment was from Bio-Rad Laboratories (Richmond, CA, USA). The fluorogenic ImaGene green C_12_FDG substrate reagent and CellROX deep red probe for oxidative stress detection were from Molecular Probes (Thermo Fisher Scientific brand, Madrid, Spain). Trizol reagent and RNA later solution was from Ambion-Life technologies (Thermo Fisher Scientific brand, Madrid, Spain). Protease inhibitor cocktail tablets and FastStart universal probe master were from Roche Diagnostics S.L. (Barcelona, Spain). High capacity cDNA reverse transcription kit and TaqMan gene expression assays from mouse were purchased from Applied Biosystems (Thermo Fisher Scientific brand, Madrid, Spain). AKT inhibitor II was from Merk Millipore (BioNova científica, Madrid, Spain). Tirofiban hydrochloride (Agrastat) was from Correvio Spain S.L.U. (Madrid, Spain). Human ET-1, Fibronectin 0.1% solution, antagonists from endothelin receptors such as BQ123 for ET_A_, BQ788 for ET_B_, and Bosentan hydrate for ET_A_ and ET_B_, antagonist from phosphatidylinositol 3-kinase (PI_3_K), such as LY-294,002 hydrochloride (LY) and Wortmannin (WTN), as well as the rest of drugs, antibodies and reagents (unless otherwise indicated) were from Sigma-Aldrich-Fluka Chemical Co. (St. Louis, MO, USA).

### Cell culture

C_2_C_12_, a mouse myoblast cell line, was purchase from American Type Culture Collection (Manassas, VA, USA). Cells were grown in Dulbecco’s Modified Eagle Media (DMEM) containing 4.5 g/L glucose and supplemented with 10% fetal bovine serum, 100 U/mL penicillin and 100 μg/mL streptomycin in an atmosphere of 95% air and 5% CO_2_. Cells were used always at passages under 15.

### Experimental design

To determine whether ET-1 induces senescence and fibrosis in C_2_C_12_, cells were incubated with 1 nM ET-1 at different times. The choice of ET-1 dose was based on previous works [58]. To study the mechanisms implied, we used several antagonists: 1) To block endothelin receptors a dual ET_A/B_ receptor antagonist (Bosentan, 10 μM), a specific ET_A_ receptor antagonist (BQ123, 100 nM) or a specific ET_B_ receptor antagonist (BQ788, 100 nM) were used. 2) To block ROS production the antioxidant N-acetylcysteine (NAC, 100 μM) was used. 3) To block PI_3_K-AKT-GSK pathway we used wortmannin (WTN, 10 μM) and LY-294,002 hydrochloride (LY, 50 μM), which block phosphatidylinositol 3-kinase (PI_3_K) preventing phosphorylation and activation of AKT; and also the AKT inhibitor (I-AKT, 30 μM), which blocks phosphorylation of GSK-3ß.

### Animal studies

Male C57BL6 mice from different ages: 5 (n=20), 18 (n=10), and 24 (n=20) month-old were obtained from Janvier Laboratories. Animals were kept on a 12:12h light-dark cycle, at 24°C, and food and water were available ad libitum. Mice were anesthetized and blood samples were collected by heart puncture exsanguinations. The gastrocnemius and the tibialis anterior muscles were isolated, one part was conserved in RNA later solution for protein extraction and other part was frozen on OCT to histological studies. Serum ET-1 was measured by enzyme-linked immunosorbent assay (ET-1 ELISA) according to the kit instructions (IBL Co.) using a 96-well microplate reader.

### Grip strength test

To measure 4 limb grip strength, we used a Grip Strength Meter (UGO BASILE) from PSYMTEC (Madrid, Spain). The procedure was as follows: grip strength was measured by gently pulling the mouse by the tail in a horizontal plane parallel with the base plate of the grip strength meter. The animal’s grip strength was measured as peak force registered. Each mouse was tested ten times consecutively per day at one-minute intervals, with the maximum grip strength used in analyses. The order of mice tested by this test was randomized. All investigators were blinded to all results. Test sessions were performed during the afternoon hours of the light cycle (11 AM to 2 PM).

### Sirius red staining

Gastrocnemius and tibialis anterior muscles were frozen immediately on OCT after being isolated. Tissues were cut and stored at -80°C until the assay was performed. Tissues were stained using the Picro Sirius Red Stain kit according to the manufacture instructions for 10 min. After that, they were dehydrated and mounted with DPX solution to be observed with a microscope. Pictures were obtained with 20x magnification and Sirius red intensity was measured using Image Pro Plus software (http://www.mediacy.com/imageproplus).

### Western blot assays

Proteins were obtained from cells or mice muscles by using the Lysis Buffer (20 mM Tris-HCl pH 7.5, 150 mM NaCl, 1 mM EGTA, 1 mM EDTA, 0.1 % sodium deoxycholate, 1 % Triton X-100, 10 mM sodium pyrophosphate) containing a protease inhibitor cocktail. The resulting solution was spun at 13,000 rpm for 30 min at 4°C. Protein concentration was determined with BioRad protein assay kit. Equal amounts of protein (30 μg protein/lane) from each sample were separated on SDS-polyacrylamide gels (PAGE) under reducing conditions and transferred onto nitrocellulose membranes. Membranes were blocked with 5% (w/v) non-fat dry milk in Tween Tris buffered saline (TTBS) (20 mM Tris-HCl pH 7.5, 0.9% NaCl, 0.05% Tween 20) for 1h at room temperature (R/T), and then incubated with different specific antibodies for FN or p16 detection. Rabbit monoclonal anti-p16 antibody was incubated for O/N at 4°C (1:1000 dilution in TTBS with 3% BSA), and rabbit polyclonal anti-FN was incubated for 1h at R/T (1:1000 dilution in TTBS with 0.05% BSA). After washing in TTBS, blots were incubated for 1h at R/T with horseradish peroxidase-conjugated goat anti-rabbit IgG (10,000-fold diluted for FN and p16), as secondary antibody. The immunoreactive bands were visualized with the SuperSignal West Pico detection system after 30 sec of exposure to CL-Xposure films. Then, blots were reblotted with a rabbit anti-actin antibody in order to normalize FN or p16 levels in cells, or mouse anti-GAPDH antibody in mice.

Similar experiments were performed in myoblasts to study whether the ET-1 effect on fibrosis was mediated by activation of PI_3_Kinase-AKT-GSK pathway. Proteins were separated on 6% SDS-PAGE and phosphorylation of AKT and GSK were detected by immunoblot using antibodies against P-AKT or P-GSK-3ß, comparing these changes with total AKT and total GSK expression. Blots were incubated 2h at R/T with 1:1000 dilution of each antibody in TTBS with 0.05% BSA. After washing in TTBS, blots were incubated for 1h at R/T with 1/10,000 horseradish peroxidase-conjugated goat anti-rabbit IgG. The immunoreactive bands were visualized with the SuperSignal West Pico detection system after 30 sec of exposure to MXB film. Then, blots were reblotted with a rabbit anti-actin antibody in order to normalize P-AKT and P-GSK levels.

To test ET_A_ or ET_B_ receptor expression in C_2_C_12_, we compared its expression with the one in endothelial cells, using two specific anti-rabbit antibodies by Western blot, which were incubated 2h at R/T with 1:1000 dilution. Then, blots were normalized using actin antibody.

### Detection of senescence-associated ß-galactosidase activity by fluorescence confocal microscopy

C_2_C_12_ myoblast cells were grown in microscope cover glasses and after 24h of free-serum DMEM, they were treated with 1 nM ET-1 at different times, in the presence or not of different antagonists. In some cases, cells were also treated with 2.5 μg/ml FN at different times. To determine cellular senescence, senescence-associated ß-galactosidase (SA-ß-GAL) activity was measured by fluorescence confocal microscopy, using the fluorogenic substrate C_12_FDG [35]. After treatments, 33 μM C_12_FDG was added and incubated for 2h. At the end of incubation, cells were washed twice with PBS, and fixed with 4% para-formaldehyde for 15 min. Subsequently, cells were washed again and mounted in ProLong® Gold antifade reagent with DAPI overnight. Samples were analyzed using LEICA TCS-SP5 confocal microscope (Leica Microsystems; GmbH, Mannheim, Germany) at 488 nm argon laser to detect green fluorescence of SA-ß-GAL activity and at 405 nm to detect DAPI. Pictures were obtained and fluorescence intensity was measured by densitometry by Image J software (http://rsbweb.nih.gov/ij/).

### Quantitative RT-PCR

Total RNA from C_2_C_12_ myoblast cells were isolated using Trizol reagents according to the manufacturer’s protocol. cDNA was synthesized using a High Capacity cDNA reverse transcription kit [28], and FN and β-Actin expression were measured by quantitative PCR (ABI Prism 7500 Fast Real-Time PCR System) and analyzed with 7500 Fast sequence detection software v1.3.1 (Applied Biosystems Inc., Foster City, CA, USA), using Taqman genes and Double delta Ct method. Taqman genes: FN (Mm01256744_m1), and the endogenous control beta-Actin (Mm61205647_g1) were used.

To test ET_A_ or ET_B_ receptor expression in C_2_C_12_, we compared its expression with the one in endothelial cells, using two specific Taqman genes: ET_A_ receptor (Mm01243722_m1, Ednra), ET_B_ receptor (Mm00432989_m1, Ednrb). To test pro-inflammatory cytokines expression in C_2_C_12_, we used the specific Taqman genes: IL-6 (Mm00446190_m1, Il6), TNF-alfa (Mm00443258_m1, Tnf) and MCP-1 (Mm00441242_m1, Ccl2).

### Cellular localization of FN by immunofluorescence

Cells were grown on cover slips for 24h. ET-1 was added in the presence or in the absence of different antagonists. After being washed twice, cells were fixed with 4% p-formaldehyde for 10 min at R/T, then 0.5% Triton X-100 was added and incubated for 10 min at R/T. After that, cells were blocked with 5% BSA for 1h at R/T, and then incubated overnight at 4°C with mouse anti-FN (1:100). After being washed in PBS, cells were incubated with 200-fold diluted goat anti-mouse IgG labeled with Alexa Fluor 647. Cover slips were mounted in Prolong Gold antifade reagent with DAPI to stain nuclei. Samples were analyzed using LEICA TCS-SP5 confocal microscope (Leica Microsystems; GmbH, Mannheim, Germany) at 633 nm helium-neon laser to detect red fluorescence of FN antibody and at 405 nm to detect DAPI. Pictures were obtained and fluorescence intensity was measured by densitometry by Image J software (http://rsbweb.nih.gov/ij/).

### ROS production

C_2_C_12_ myoblast cells were grown in 60μ-dishes (35mm high) with glass bottom (Ibidi, Martinsried, Munich, Germany) and after 24h of free-serum DMEM, cells were treated at different times with ET-1 in the presence or not of the antioxidant NAC. ROS production was measured by fluorescence confocal microscopy, using the CellROX Deep Red probe. After ET-1 treatment, 5 μM CellROX was added and incubated for the last 30 min. At the end of incubation, cells were washed twice with PBS, and fixed with 4% para-formaldehyde for 15 min. Cells were analyzed using LEICA TCS-SP5 confocal microscope (Leica Microsystems; Wetzlar, Germany) at 633 nm helium-neon laser to detect red fluorescence of CellROX probe. Pictures were obtained and fluorescence intensity was measured by densitometry by Image J software (http://rsbweb.nih.gov/ij/).

### Transfection of siRNA against ILK

C_2_C_12_ myoblast cells were grown until they reached 60-80 % confluent. After that, siRNA against ILK or scrambled control from Santa Cruz Biotechnologies (Quimigen, Madrid, Spain) were transfected using Lipofectamin 2000 for 16h in OptiMEM medium at 37°C. Cells were washed twice with PBS before growing in DMEM with serum for 24h. After that, 2.5 μg/ml FN was added for 48h to assess p16 expression after checking the silencing of ILK by western blot.

### Statistical analysis

GraphPad Prism 5 Software was used for statistical analysis. The following statistical tests were applied in cells experiments: one-way ANOVA followed by Dunnett´s post-tests for experiments compared with control cells, or followed by Bonferroni post-tests for multiple comparisons. Statistical tests applied in experiments performed on animals were: one-way ANOVA followed by Bonferroni post-tests for multiple comparisons, and t-test followed by unpaired t test with Welch's correction for two groups comparisons. Correlations were analyzed using the non-parametric test Spearman correlation. Unless otherwise specified, data are expressed as the mean ± standard error, and expressed as a percentage of the control values of a variable number of experiments detailed in figure legends. The level of statistically significance was defined as p < 0.05.

### Ethical statement

The study design and the animal experimental protocols were performed in agreement with the Guide for the Care and Use of Laboratory Animals published by the US National Institute of Health (NIH Publication No.85-23, revised 1996) and with the European Union regulations (EU Directive 2010/63/EU). The study was revised and approved in accordance with the Ethics Committee from Alcala University (Madrid, Spain). Animal works were taken in the breeding place of Alcala University.

## Supplementary Material

Supplementary Figure 1
