# Supplementary material for: Endothelin-1 induces cellular senescence and fibrosis in cultured myoblasts. A potential mechanism of aging-related sarcopenia
Source: Aging (Albany NY). 2020 Jun 22;12(12):11200–23. doi: 10.18632/aging.103450 (PMC7343454; doi:10.18632/aging.103450)
Supplement: Supplementary Figure 1 [file aging-12-103450-s001..pdf]

## SUPPLEMENTARY FIGURES

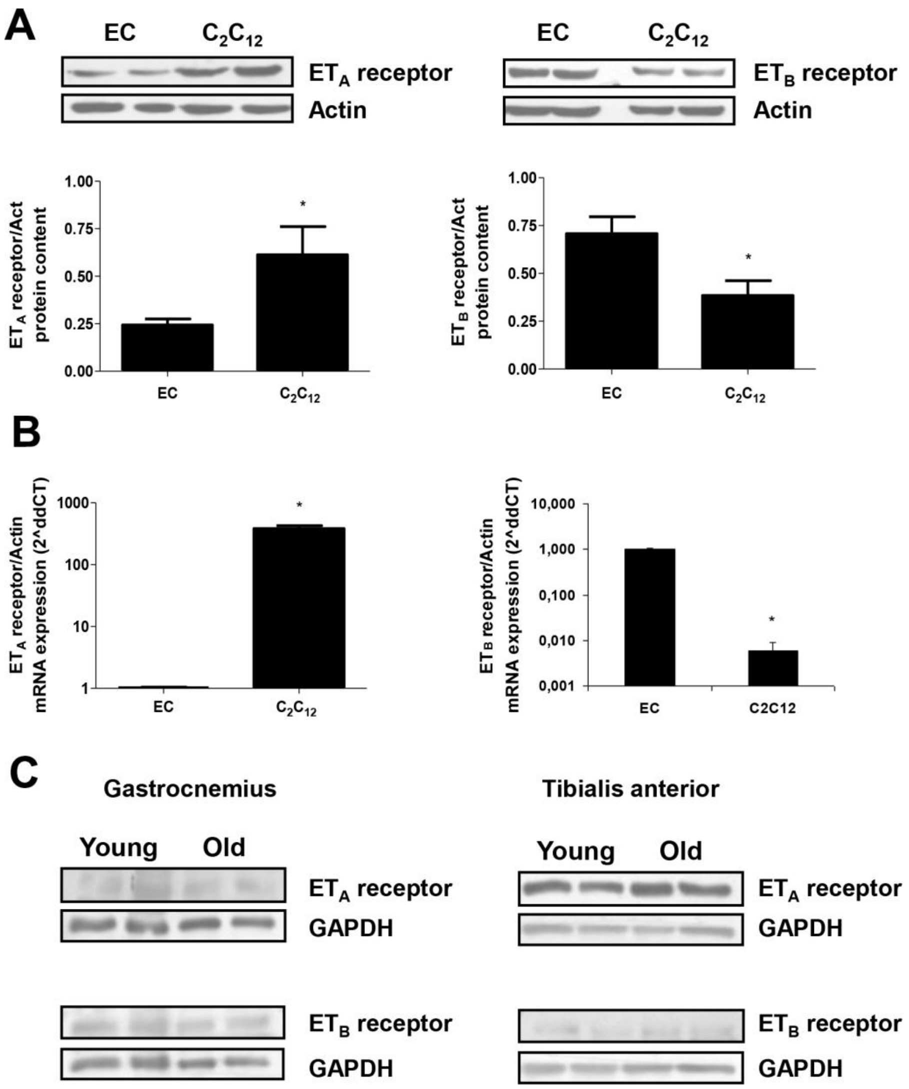


**Supplementary Figure 1. Expression of ET receptors in mouse myoblasts ($C_2C_{12}$) and gastrocnemius and tibialis anterior muscles from mice.** Expressions of $ET_A$ and $ET_B$ receptors were assessed in cultured cells, endothelial cells (EC) and mouse myoblasts ($C_2C_{12}$) (**A**, **B**), and mice muscles (**C**), by Western blot (**A**, **C**) and by RT-qPCR in cells (**B**). Representative Western blots are shown at the top of panel A with the densitometric analyses below. Values are the mean±SEM of 3 independent experiments, *p<0.05 vs. endothelial cells (EC).

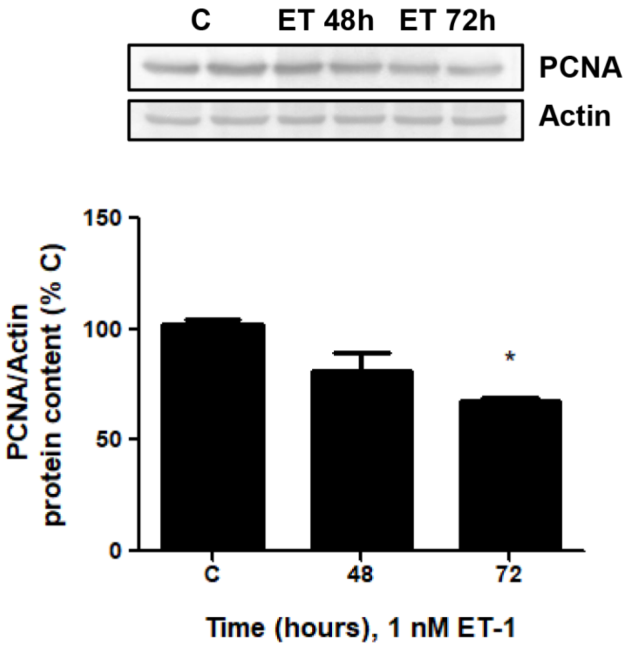


**Supplementary Figure 2. Endothelin-1 reduces PCNA expression in mouse myoblasts ($C_2C_{12}$).** Cells were incubated with 1 nM ET-1 at different times. Then, PCNA protein content was assessed by Western blot. A representative Western blot is shown at the top with the densitometric analysis below. Values are the mean±SEM of 3 independent experiments, *p<0.05 vs. control cells (C).

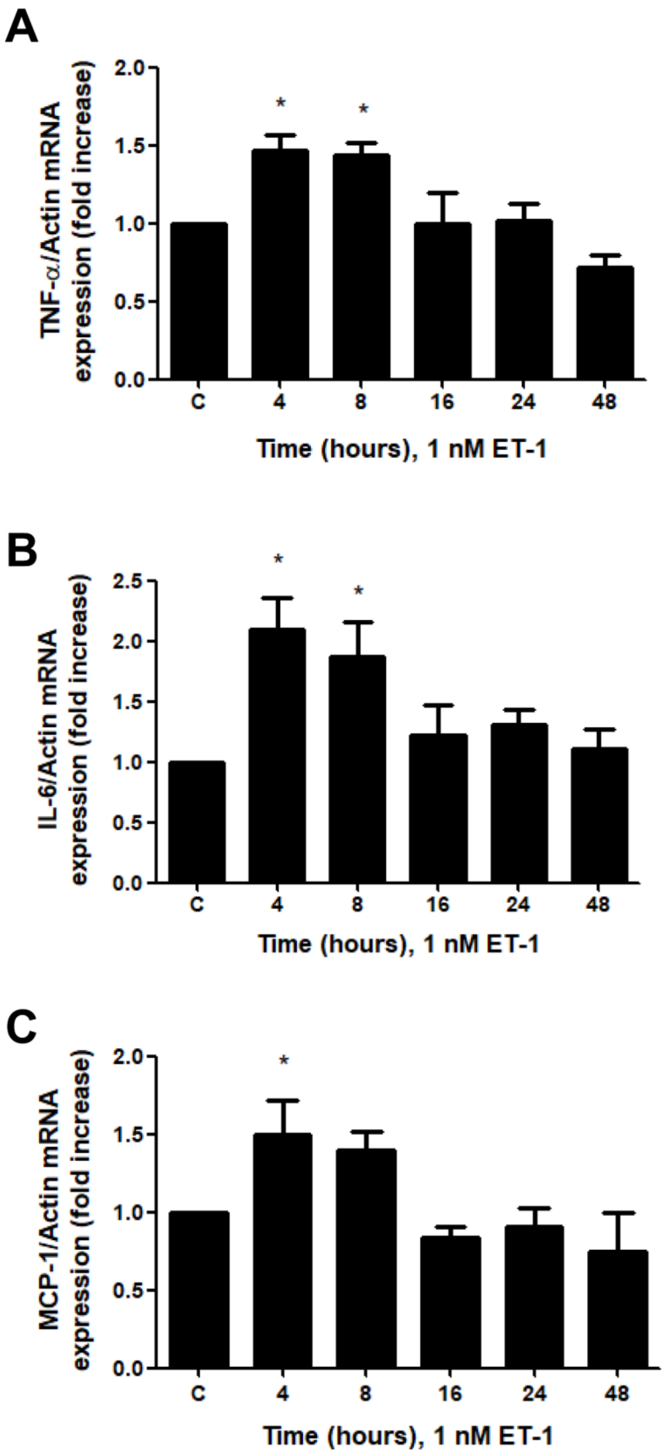


**Supplementary Figure 3. Endothelin-1 increases pro-inflammatory cytokines expression in mouse myoblasts ($C_2C_{12}$).** Cells were incubated with 1 nM ET-1 at different times. Then, TNF-α (**A**), IL-6 (**B**) and MCP-1 (**C**) mRNA expressions were assessed by real-time PCR. Values are the mean±SEM of 3 independent experiments, *p<0.05 vs. control cells (**C**).
